# Twelve-year outcomes of bedside laser photocoagulation for severe retinopathy of prematurity

**DOI:** 10.3389/fped.2023.1189236

**Published:** 2023-06-23

**Authors:** Bingzhi Yang, Chaohui Lian, Ruyin Tian, Yi Chen, Song Tang, Haishan Xiang, Honghui He, Guoming Zhang

**Affiliations:** ^1^ Department of Eye Care, Affiliated Shenzhen Maternity & Child Healthcare Hospital, Southern Medical University, Shenzhen, China; ^2^ Shenzhen Eye Hospital, Jinan University, Shenzhen Eye Institute, Shenzhen, China

**Keywords:** anesthesia, bedside, laser photocoagulation, low birth weight, neonatal intensive care unit, premature, retinopathy of prematurity (ROP), sedation

## Abstract

**Purpose:**

The purpose of this study is to evaluate the 12-year outcomes of bedside laser photocoagulation (LP) for severe retinopathy of prematurity (ROP) under sedation combined with ocular surface anesthesia in neonatal intensive care units (NICU).

**Design:**

The study is a retrospective case series.

**Methods:**

Infants treated with bedside LP for severe ROP from April 2009 to September 2021 were included. All LP treatments were performed under sedation and surface anesthesia at the bedside in NICU. Data were recorded for clinical and demographic characteristics, total laser spots, duration of treatment, proportion of total regression of ROP, proportion of recurrence, and adverse events.

**Results:**

A total of 364 infants (715 eyes) were included, with a mean gestational age of 28.6 ± 2.4 weeks (range: 22.6–36.6 weeks) and a mean birth weight of 1,156.0 ± 339.0 g (range: 480–2,200 g). The mean number of laser spots was 832 ± 469, and the mean duration of treatment was 23.5 ± 5.3 min per eye. Of all the eyes, 98.3% responded to LP with complete regression of ROP. ROP recurred in 15 (2.1%) eyes after the initial LP. Additional LP was performed in seven (1.0%) eyes. No patient exhibited mistaken LP of other ocular tissues, and there were no serious ocular adverse effects. None of them needed endotracheal intubation.

**Conclusions:**

Bedside LP treatment is effective and safe for premature infants with severe ROP under sedation and surface anesthesia in NICU, especially for infants whose general condition is unstable and not suitable for transport.

## Introduction

Retinopathy of prematurity (ROP) is a common blinding eye disease in infants with premature age or low birth weight (BW) (1, 2). At present, early screening and timely intervention are the main approaches to avoid childhood blindness or low vision caused by ROP, while laser photocoagulation (LP) remains the most common treatment modality for severe ROP (3–7).

Many different methods for the administration of anesthesia and perioperative management of severe ROP laser therapy have been developed (8–15). In most cases, an infant with severe ROP is transported to the operating room for laser treatment with general anesthesia. Although general anesthesia has a reliable analgesic effect, it causes tracheal intubation and mechanical ventilation, which can lead to lung injury (16), thereby worsening clinical symptoms of bronchopulmonary dysplasia (BPD), increasing the risk of pulmonary infection, and even causing the development of life-threatening postoperative apnea (PA) (17). Moreover, severe ROP mostly occurs in very preterm and very low birth weight infants (1). Some preterm infants are still systemically unstable when laser therapy for ROP is required. The transport of these infants carries inherent risks (18). To provide safe transfer, a highly qualified transport team, a sophisticated incubator, vehicular first-aid devices, and an ambulance are required (19), which is obviously inconvenient.

To avoid the inconvenience and risk of general anesthesia and transport, we performed LP treatments on premature infants with severe ROP under intravenous sedation of diazepam and phenobarbitone combined with ocular surface anesthesia at the bedside in the neonatal intensive care unit (NICU) since April 2009. As we previously reported, diazepam and phenobarbitone combined with surface anesthesia appears to be a safe and effective alternative anesthesia for LP treatment for severe ROP (20). The pilot study also published data relating to CRIES (C: crying; R: requires increased oxygen administration; I: increased vital signs; E: expression; S: sleeplessness) pain assessment, comparison of microblood glucose before and after operation, and intraoperative and postoperative complications (20). In this paper, we report the efficacy of bedside LP treatment under sedation and surface anesthesia for severe ROP in NICU from April 2009 to September 2021.

## Materials and methods

### Participants

This study was a single-center retrospective case series. It was conducted at the NICU of the Shenzhen Maternity and Child Healthcare Hospital, which is one of the largest perinatology centers in Shenzhen city, China. A total of 364 infants who underwent indirect diode LP for severe ROP between April 2009 and September 2021 were included.

All LP treatments were performed under sedation and surface anesthesia at the bedside in NICU. The parents of the infants provided written informed consent for the collection and use of their child’s clinical information for research purposes before inclusion in the study. The hospital Ethics Committee approved this study.

The inclusion criteria included (1) having gestational age at birth of ≤34.0 weeks or birth weight of <2,000 g, (2) being diagnosed with ROP, (3) meeting the indications for retinal LP treatment, (4) having undergone retinal LP treatment, and (5) having a follow-up for more than 1 year after LP.

The exclusion criterion was the presence of any ocular diseases apart from ROP.

### Methods

Under topical anesthesia after mydriasis, an ophthalmologist with screening experience in ROP used a binocular indirect ophthalmoscope for examination at the bedside in NICU. For infants with severe ROP, a neonatal digital wide-field fundus imaging system (RetCam3) was used for fundus photography before LP treatment.

The procedures for diagnosis and treatment were in accordance with previously described screening guidelines for ROP (21–23). Briefly, the initial examination time was either 4–6 weeks after birth or 31 weeks postmenstruation, whichever came later, while follow-up schedules and treatment decisions were in accordance with the indications proposed through the Early Treatment for Retinopathy of Prematurity study results (24–26). In addition, for the threshold stage or type I prethreshold lesions, except zone 1 ROP and aggressive ROP (A-ROP), LP was performed within 72 h from diagnosis. According to the latest treatment concepts for ROP (27, 28), anti–vascular endothelial growth factor (anti-VEGF) treatment was performed first for zone 1 ROP and A-ROP, while the laser therapy was performed for relapsing patients.

Neonatologists and ophthalmologists jointly evaluated treatment risks and determined treatment protocols. Firstly, oxygen channels, resuscitation bags, suitable masks, and endotracheal devices were prepared before surgery. Then, the infant was subjected to 3 h of fasting, all oral medications were stopped, and intravenous access was established to prevent hypoglycemia. One hour before the operation, tropicamide phenylephrine eye drops (Mydrin-P) were administered once every 15 min to dilate pupils, for a total of four times. The pupils were dilated to more than 6 mm in diameter. Thereafter, the infant was administered with an injection of phenobarbitone, 30 min prior to LP treatment, at a dosage of 10 mg/kg, while diazepam was injected 5 min prior to the treatment at a dosage of 0.25–0.49 mg/kg. Proparacaine hydrochloride eye drops (Alcaine) were administered 5 min before and immediately before the treatment per eye. If the operation took more than 15 min, proparacaine hydrochloride eye drops were administered again. During the operation, if the infant had frequent physical movements, crying, or painful expressions, intravenous diazepam 0.2 mg/kg was administered. The ophthalmologist prepared an infant eyelid opener, scleral press, +20 D aspheric lens, binocular indirect lens laser output system, and 810 nm infrared laser machine. The French BVI Kota company manufactured the laser adapter, while the binocular indirect ophthalmoscope was from the German HEINE company.

Experienced pediatric ophthalmologists performed all LP treatments in the NICU. Infants were transferred from their incubator and placed on a neonatal warming medical rescue table. During surgery, the neonatologist held the infant, observed the vital signs, prepared for rescue, and, if necessary, administered oxygen or cardiopulmonary resuscitation. Next, the ophthalmologists used +20 D aspherical lenses to assist binocular indirect ophthalmoscopes, then used an 810 nm infrared laser to perform LP to the peripheral avascular retina. Scleral pressure technology was adopted for the operation of the far peripheral retina. LP was performed on avascular retina from the edge of the ridge to the edge of the ora serrata. The laser wavelength was 810 nm, with an energy of 110–250 mW, an initial energy of 110 mW, and an exposure time of 200–250 ms. A semi-fusion spot (half spot width apart from each other) and a grade III light spot intensity (the retina produces a gray-white reaction) were also used. Cornea moisture was maintained during the treatment procedure.

Continuous monitoring of the heart rates, respiratory rates, and oxygen saturation was conducted throughout the treatment period. Apnea was defined as cessation of breathing ≥20 s. Bradycardia was defined as heart rate <60 beats per minute. Hypotension was defined as mean arterial pressure <45 mm Hg. The duration of the treatment procedure was defined as the time required to perform LP per eye and was calculated by subtracting the stop time from the start time.

After the treatment, the heart rate, respiratory rate, and oxygen saturation was continuously monitored. In addition, vital signs were observed and blood sugar was measured, while oral feeds were appropriately administered 6 h after surgery. Postoperative ocular medication was administered: tropicamide phenylephrine eye drops Qid, tobramycin and dexamethasone eye drops Qid, pranoprofen eye drops Qid, and tobramycin and dexamethasone eye ointment Qn were given in the first week after surgery. Ophthalmology follow-ups were performed at 1 week, 1 month, 2 months, 3 months, 6 months, 9 months, and 1 year after LP treatment to evaluate the curative effect and any associated complications. RetCam3 photographs were taken during the evaluation.

ROP regression was defined as the involution of neovascularization and the growth of retinal vessels passing through the atrophic retina without plus disease (29, 30). Total regression was defined as the ROP regressed after LP, including initial and additional treatment. Unresponsiveness was considered as the absence of any regression of neovascularization and the plus disease in the first postoperative week (31, 32). The reappearance of neovascularization and plus disease was evaluated as recurrence (reactivation of ROP). An unsatisfactory anatomic outcome was considered as the presence of any of the following: total or partial retinal detachment, localized tractional or non-tractional membranes at the posterior pole or in the retinal periphery, and dragging of the disc (31).

### Statistical analysis

Statistical analyses of basic demographic and clinical characteristics were performed using SPSS 22.0. Continuous variables were presented as means ± standard deviation, while categorical variables were presented as numbers (*n*) and percentages (%).

## Results

Table 1 presents a summary of the demographic and preoperative characteristics of the infants.

**Table 1:** Demographic and preoperative characteristics of the infants.

Clinical characteristics	Value
Total cases, *n* (eyes)	364 (715)
Gender, *n* (%)	
Male	231 (63.5)
Female	133 (36.5)
GA (week), (mean ± SD)	28.6 ± 2.4
Birth weight (g), (mean ± SD)	1,156.0 ± 339.0
Postmenstrual age (week), (mean ± SD)	38.5 ± 3.1
Definition of the baby by birth week, *n* (%)	
<24 weeks	5 (1.4%)
<28 weeks EPI	141 (38.7%)
28 ≤ GA < 32 weeks	186 (51.1%)
32 ≤ GA < 34 weeks	33 (9.1%)
34 ≤ GA < 37 weeks	9 (2.5%)
Definition of baby by birth weight, *n* (%)	
<500 g	1 (0.3%)
<750 g ILBW	43 (11.8%)
<1,000 g ELBW	85 (23.4%)
1,000 ≤ BW < 1,500 g VLBW	164 (45.1%)
1,500 ≤ BW < 2,000 g LBW	66 (18.1%)
2,000 ≤ BW < 2,200 g LBW	5 (1.4%)
Classification of ROP, eyes (%)	
Threshold stage ROP	467 (65.3%)
Type 1 prethreshold ROP	248 (34.7%)

GA, gestational age; EPI, extremely preterm infants; ILBW, incredibly low birth weight; ELBW, extremely low birth weight; VLBW, very low birth weight; LBW, low birth weight.

All bedside laser treatments were successfully completed. Table 2 outlines the duration of operation, laser spots, and intraoperative complications. Forty-three patients (11.8%) were suspended from the operation due to bradycardia, including four patients (1.1%) with apnea. After 2–3 min of positive pressure ventilation with a mask, all vital signs recovered, and the operation continued. None of them needed endotracheal intubation.

**Table 2:** Duration of operation, laser spots, and intraoperative complications.

Variables	Value
Duration of operation (min) (mean ± SD) (per eye)	23.5 ± 5.3
Laser spots (points) (mean ± SD) (per eye)	832 ± 469
Intraoperative complications, *n* (%)	
Apnea, *n* (%)	4 (1.1%)
Tracheal intubation, *n* (%)	0
Bradycardia, *n* (%)	43 (11.8%)
Mask positive pressure ventilation, *n* (%)	43 (11.8%)

Table 3 shows a summary of treatment outcomes. ROP completely regressed in 703 (98.3%) eyes after LP treatment, in which 696 (97.3%) eyes regressed after the initial LP and 7 (1.0%) eyes after additional LP. Unresponsiveness was noted in 4 (0.6%) eyes in the first postoperative week, and intravitreal bevacizumab treatment was performed. Recurrence was noted in 15 (2.1%) eyes in the first postoperative week to the second postoperative month, of which 7 (1.4%) eyes were treated with additional LP, 5 eyes with intravitreal bevacizumab treatment, and 3 (0.4%) eyes with vitrectomy. No unsatisfactory anatomic outcome was found in all cases after a year of postoperative follow-up.

**Table 3:** Treatment outcomes.

Variables (eyes) (%)	Value
Total ROP regression	(703) 98.3%
Regression after initial LP	(696) 97.3%
Regression after additional LP	(7) 1. 0%
Repeat treatment	(19) 2.7%
Unresponsiveness	(4) 0.6%
Recurrence	(15) 2.1%
Additional LP	(7) 1. 0%
Intravitreal bevacizumab treatment	(9) 1.3%
Vitrectomy	(3) 0.4%

Table 4 shows a summary of postoperative complications and intensive care follow-ups. Briefly, patients exhibited no mistaken photocoagulation of other ocular tissues, and there were no serious ocular complications, such as corneal or iris injuries, anterior ischemia, cataracts, uveitis, macular burns, retinal detachment, central retinal artery occlusion, or eye infections. The subconjunctival hemorrhages were absorbed spontaneously at the 1-week follow-up, whereas retinal hemorrhages were absorbed spontaneously at the 6-month follow-up. Postoperative apnea occurred in three cases. Among them, one case recovered after pressure ventilation with a mask, and two patients needed mechanical ventilation (one case of nasal continuous positive airway pressure and one case of nasal synchronized intermittent mandatory ventilation) for 3 days. Notably, seven infants became cyanotic during feeding, within 24 h after the treatment, necessitating suspension of feeding. However, after back-patting and oxygen administration, all returned to normal. Non-infectious low-grade fever subsided spontaneously on the second day.

**Table 4:** Postoperative follow-up.

Variables	Value
Retinal hemorrhage (eyes) (%)	(35) 4.9%
Subconjunctival hemorrhage (eyes) (%)	(293) 41.1%
Non-infectious low-grade fever (*n*) (%)	(2) 0.5%
Apnea (*n*) (%)	3 (0.8%)
nCPAP (*n*) (%)	1 (0.3%)
nSIMV (*n*) (%)	1 (0.3%)
Tracheal intubation (*n*) (%)	0
Cyanosis during feeding (*n*) (%)	(7) 1.9%

nCPAP, nasal continuous positive airway pressure; nSIMV, nasal synchronized intermittent mandatory ventilation.

## Discussion

To date, the most common treatment modality for severe ROP was LP (80.7%–85.0%), followed by anti-VEGF treatment (13.3%–20.0%) (33, 34). The prevalence rate of severe ROP is highest for infants with very premature age and very low birth weight; hence, infants requiring treatment for ROP are the most vulnerable (1). Preterm infants can perceive pain, tend to be more stressed in response to painful stimuli, and are more susceptible to episodes of apnea and bradycardia (35). Thus, adequate sedation/analgesia and trauma avoidance are essential for preterm infants undergoing LP. However, no consensus currently exists regarding the safest anesthetic regimen for premature infants.

Indeed, general anesthesia is reliable and can effectively prevent wrong photocoagulation of other ocular tissues. However, perioperative risk and subsequent risk of neurodevelopmental deficits are significantly higher for premature infants than for older children and adults. Firstly, general anesthesia and mechanical ventilation have been associated with the development of lung injury in premature infants, exacerbation of BPD (36), difficulty in ventilator removal, lung infections, and even life-threatening PA (17, 37). Secondly, evidence has found an association between anesthesia exposure and cognitive, memory, listening comprehension, and language deficits (38–40). McCann et al. (41) reported that less than 1 h of general anesthesia in early infancy does not alter neurodevelopmental outcomes at the age of 5 years compared with awake-regional anesthesia, which provided the strongest and most reassuring evidence for infant exposure to general anesthesia to date. However, data regarding the influence of repeated or longer exposures to general anesthesia are less clear. Concerning a premature infant, multisystem immaturity usually creates a number of medical problems, for example, asphyxia, ROP, BPD, hyaline membrane disease, necrotizing enterocolitis, intraventricular hemorrhage, patent ductus arteriosus, deficient drug metabolism, hematologic derangements, and temperature dysregulation, and they often present to surgery. It is difficult to guarantee that they only need one surgery before the age of five and that the duration of exposure to anesthesia is within 1 h. In addition, many infants treated with LP under general anesthesia should be transferred to a hospital with on-site pediatric anesthesia and laser machines. Infants undergoing severe ROP are frequently unwell and usually suffer from several complications of preterm delivery. The transport of these infants carries inherent risks. Mohamed et al. (18) reported the correlation of interhospital transport with intraventricular hemorrhages in very low birth weight infants from one of the largest databases in the USA, collecting data from more than 1,000 hospitals. To provide safe transfer, a highly qualified transport team, a sophisticated incubator, vehicular first-aid devices, and an ambulance are required (19). Obviously, it is also very inconvenient to transfer premature infants from the NICU of a maternity and child healthcare hospital to the operating room of an eye specialist hospital.

Over the past decade, morphine analgesia was often used as an alternative to general anesthesia during LP treatment for ROP. However, infants were usually mechanically ventilated because of morphine-induced apnea during treatments (42). Hartley et al. (43) demonstrated that although morphine is often used to sedate ventilated infants, its analgesic efficacy is unclear, and they strongly advised caution if considering its use for other acute painful procedures in non-ventilated premature infants.

After estimating complication rates, Faruk et al. (44) suggest that fentanyl may be safer than morphine in laser surgery for ROP. Piersigilli et al. (8) reported that sedation with propofol and fentanyl for LP treatment could prevent intubation and mechanical ventilation. Nevertheless, the Food and Drug Administration does not recommend the use of propofol in babies younger than 3 years old. Recently, remifentanil has been suggested as an alternative to propofol and fentanyl for infants in laser surgery for ROP (45). However, all neonates required intubation during the surgery.

Ketamine has also been recently used for neonatal sedation. It provides anesthetic, analgesic, sedative, and bronchodilatation effects, avoiding intubation. Lyon et al. (46) reported that ketamine sedation allows laser surgery to be performed in the NICU setting and avoids the potential risk of general anesthesia and inter- and intra-hospital transfer. Recent research showed that ketamine sedation could be used in premature infants with few perioperative complications and provide satisfactory conditions for the treatment of ROP (47). However, previous studies raised concerns about its use in preterm neonates as it can be associated with neurotoxicity. The latest study suggested that previous animal studies demonstrating the neurotoxicity of ketamine had unclear human translatability, and evidence supporting it in humans is lacking (48). Nevertheless, the ketamine technique should be provided by an experienced pediatric anesthetist, with resource implications.

In this study, intravenous sedation combined with ocular surface anesthesia did not require general anesthesia and intubation and allowed laser surgery to be performed only by an ophthalmologist and a neonatologist, without any pediatric anesthetist. All procedures were performed in the NICU setting, avoiding inter- and intra-hospital transfer.

Various studies have reported the efficacy rate of LP in treating ROP ranging from 82.1% to 98.0% (49–53). In the current study, complete ROP regression was seen in 696 (97.3%) of 715 eyes after initial LP and in 703 (98.3%) of 715 eyes after initial and additional LP treatment, while ROP recurred in only 19 (2.7%) of 715 eyes; these results favorably compare with other previous studies. Sizmaz et al. (49) reported treating 207 eyes with zone 2 ROP using 532 nm neodymium-doped yttrium aluminum garnet (Nd:YAG) laser, under general endotracheal anesthesia in the operation room, and ROP regressed in 96.8% of the eyes. Another study (50) reported treating 31 eyes with threshold ROP, using 532 nm Nd:YAG laser, under local anesthetic, and ROP totally regressed in 96.7% of the eyes, including 22.6% of eyes requiring repetitive LP. A randomized clinical trial (52) found a treatment success rate of 84.9% in 26 infants with zone 2 ROP using 810 nm diode LP, and 84.7% of infants were treated under general anesthesia. Although Uparkar et al. (53) reported favorable outcomes in up to 49/50 (98%) eyes treated with 810 nm diode laser of threshold ROP, they did not illustrate which sedation/analgesia was used for the infants. In the study of Hwang et al. (54), infants were intubated and sedated when LP was performed, and 26 (81%) of 32 eyes had complete ROP regression with favorable anatomical outcomes, while 1 (3%) of 32 eyes underwent recurrence.

In our study, the mean duration of the LP treatments was 23.5 ± 5.3 min per eye; this was comparable with that of previous studies (52, 55). Stahl et al. (52) reported a longer mean duration (64.1 min per eye) in their smaller cohort (26 infants), of which 84.7% of infants were treated under general anesthesia. In the trial of Saylan et al. (55), midazolam 0.1 mg/kg and ketamine 1 mg/kg were intravenously administered to all patients for sedoanalgesia, and they achieved a shorter mean duration (36.2 ± 10.1 min per patient). However, the transport of their patients from the NICU to the operating room, which was performed by a pediatric physician using a mechanical ventilator transport incubator, also took time and manpower.

There are some limitations to this study. The first limitation is the absence of a randomized comparison of LP treatment at the bedside with LP treatment in the operating room. The retrospective nature of the study introduced this limitation. This paper only retrospectively describes the outcomes of bedside LP treatment for ROP for the past 12 years. The second limitation is the absence of a randomized comparison of sedation/anesthetic procedures with general anesthesia for premature infants. It was not a comparative evaluation study. A previous study (20) had shown that the effect of sedation combined with local anesthesia was reliable. A CRIES score equal to or greater than four indicates that the patient was in pain (56). The average value of maximum scores in our previous study (20) was 1.90 ± 1.18, which was significantly less than four. The third limitation is the absence of pain scoring during the surgery. The pain scores from a previous study (20) had shown reliable sedation and anesthetic effects, and this study focused on evaluating surgical outcomes. Nevertheless, based on data from a large sample of more than 12 years, this study makes a useful contribution to literature with respect to the LP treatment of ROP.

As a result, bedside LP treatment under sedation combined with ocular surface anesthesia in NICU is safe and efficacious and appears to be the most practical for infants with severe ROP whose general condition is unstable and thus are not suitable for transfer.

## Data Availability

The original contributions presented in the study are included in the article, further inquiries can be directed to the corresponding author.
